# Preparation and Performance of H-PDMS/PMHS/OTS Hybrid Nanosilica Hydrophobic and Self-Cleaning Coatings on Phosphogypsum Surface

**DOI:** 10.3390/polym15173574

**Published:** 2023-08-28

**Authors:** Guang Yang, Zhonghua Chen, Changwei Lv, Lei Deng, Xiaofeng Luo, Yi Li, Songtao He, Qibin Liu

**Affiliations:** 1 College of Materials and Metallurgy, Guizhou University, Guiyang 550025, China; yg0811@foxmail.com (G.Y.);; 2 Guizhou Phosphating Green Environmental Protection Industry Co., Ltd., Guiyang 551100, China; 3 KZJ New Materials Group Guizhou Co., Ltd., Longli 551206, China

**Keywords:** phosphogypsum, siloxane, nanosilica, coatings, hydrophobicity

## Abstract

Hemihydrate phosphogypsum, an industrial solid waste product of phosphoric acid production, is abundant and inexpensive. If the problem of poor water resistance is solved, this material could be substituted for cement and other traditional energy-consuming cementitious materials in the construction industry. This approach would confer important economic and environmental benefits while promoting the resource utilization of phosphogypsum (PG). In this study, hydrophobic and self-cleaning coatings of H-PDMS/PMHS/OTS hybrid nanosilica were prepared on a post-hydroxylated PG surface using sol–gel and impregnation methods. The water contact angle, Fourier-transform infrared spectroscopy, Three-dimensional surface morphology and roughness analysis, X-ray photoelectron spectroscopy, scanning electron microscopy, surface abrasion tests, and tape adhesion tests were used to evaluate the hydrophobicity of the coatings. The results demonstrated that the in situ reaction produced a hydrophobic siloxane/nanosilica hybrid network that bonded to the PG surface via hydrogen bonding, making the otherwise completely hydrophilic PG hydrophobic (PGH-3, contact angle (CA) = 144.1°). The PGH-3 sample exhibited excellent chemical stability, maintaining a contact angle greater than 135° under strongly acidic or alkaline conditions. The contact angle remained at 123.7° after 50 tape-bonding tests. After 100 wear cycles, the contact angle remained at 121.9°. This study presents an environmentally friendly method and a straightforward application procedure to impart hydrophobicity to solid waste PG. Its potential is thus demonstrated in the field of PG-based construction materials and the comprehensive utilization of solid waste.

## 1. Introduction

Phosphogypsum (PG) is an important by-product of the phosphate compound fertilizer industry. Addressing the widespread utilization of PG resources has become an urgent issue in the phosphate compound fertilizer industry. Due to the environmental protection prevalent, utilization of PG resources has incurred significant pressure. It is therefore important to study and promote the comprehensive utilization of PG resources to achieve healthy and sustainable development of the phosphate compound fertilizer industry [1,2,3].

The gypsum industry is of considerable importance to Europe and provides a large number of jobs. The demand for construction gypsum in the EU will maintain an annual growth of 0.5–2% until 2030 [4]. The overall situation of gypsum in China shows a certain balance between supply and demand, but there is a huge demand for gypsum in localized areas due to commercial construction projects. Under the encouragement of national industrial policy, the utilization rate of industrial by-product gypsum will continue to increase in the future, and the demand for natural gypsum will tend to be stable.

Currently, more than 280 million tons of phosphogypsum every year in the world is currently being discarded in coastal areas or elsewhere and stored at production sites as stockpiles, with about 80 million tons emitted in China. In 2021, China’s by-product phosphogypsum output is about 80 million tons, and utilization is about 36.5 million tons (including mine filling), with a comprehensive utilization rate of about 45.6% [5,6].

Although phosphogypsum contains impurities such as phosphates, organic matter, fluoride, and radioactive elements, recycling phosphogypsum as a building material is a safe practice, with no further health risk from a radiological point of view [7,8]. Phosphogypsum pretreatment methods such as ultrasonic pretreatment, microwave treatment, heat treatment, chemical conditioning, and water leaching also ensure the safety of its use [9,10].

Currently, the widespread utilization of PG technology in China is based primarily on traditional low-value-added applications, such as cement retarders, gypsum building materials, and other primary utilization pathways. The lack of large-scale, large-dosing, high-value-added technology applications means that the comprehensive utilization of PG is limited by cost factors, a small radiation range, and low market capacity [6,11,12,13]. Hemihydrate PG (HPG), prepared by dehydrating raw PG slag under atmospheric pressure, exhibits high crystallinity, poor dispersion, and coarse crystals [14,15].

PG building material products are light and have acceptable heat preservation; however, the surface is hydrophilic and displays poor water resistance. Creeping and melting occur when the materials are exposed to a humid environment for a long period, resulting in a reduction in their mechanical properties. This significantly limits their large-scale industrial application in the field of functional building materials [16]. At present, the main methods for improving the hydrophobicity of PG-based materials include the addition of an internal doping-type waterproofing agent [17,18], inorganic gel material modification [19,20,21], and the preparation and application of a surface hydrophobic coating [22,23,24]. Among these, the method of preparing and applying a hydrophobic coating on the surface of PG is not only simple and efficient but also does not negatively influence the compressive and flexural strength of the PG blocks [25].

Jiang et al. [26] prepared a SiO_2_/silicone hybrid superhydrophobic coating with superhydrophobic and self-cleaning capabilities on the surface of gypsum by introducing cross-linked siloxane into the surface of the gypsum using the sol–gel method. The hydrophobic network formed by tetraethoxysilane (TEOS) reacting with hydroxy-terminated polydimethylsiloxane (H-PDMS) captures silica nanoparticles and deposits them on the gypsum crystals and interstices. Compared with untreated gypsum board, the contact angle of the treated gypsum board exceeds 150° and the sliding angle is less than 10°. Jumrus et al. [27] successfully prepared a polydimethylsiloxane (PDMS)/methyltrichlorosilane (MTCS)-modified SiO_2_-TiO_2_ NPs superhydrophobic self-cleaning coating on a commercial gypsum board. The contact angle of this coating remained greater than 150° after impact with 30,000 water drops, which demonstrates its excellent durability. The degradation rate of the coating was up to 30% when the irradiation time was one hour. Owing to the versatile surface chemistry of the polymer, its adaptability supports the excellent photocatalytic activity exhibited by the nanoparticles in the coating.

Cao et al. [28] prepared a superhydrophobic coating on a stone surface by one-step amidation. The coating exhibited high vapor diffusion, maintained the aesthetic properties of the substrate, demonstrated acceptable durability against corrosive chemicals and environmental weathering, and indicated significant potential for stone protection in rain-prone areas. Jadav et al. [29] reported a new poly(dimethylsiloxane) membrane in which hydroxyl-terminated poly(dimethylsiloxane) macromolecules were crosslinked with polymethylhydrosiloxane macromolecules and n-octadecyltrichlorosilane molecules at the same time, and the linear size of aggregates was predicted using a Debye chain model and scattering function for aggregates. It shows high potential for the treatment of organically contaminated water.

In this study, we prepared a cross-linked polysiloxane bar with multistage roughness on the PG surface through an in situ reaction and impregnation, imparting stronger hydrophobicity and lower adhesion by hybridized nanosilica. After hydroxylation, the PG surface formed hydrogen bonds with the hydroxyl groups of H-PDMS, which maintained a certain degree of hydrophobicity after several wear tests on the coating surface. The effect of the multilevel rough structure of the coating on hydrophobicity was evaluated using water contact angle testing and white-light interferometry. The action mechanism of this layer was evaluated using Fourier-transform infrared spectroscopy (FTIR), X-ray photoelectron spectroscopy (XPS), and scanning electron microscopy (SEM). The objective of this study was to optimize the performance and investigate the action mechanism of H-PDMS/PMHS/OTS hybrid nanosilica coatings for the effective utilization of solid waste PG and the functionalized application of eco-friendly and energy-saving inorganic cementitious materials.

## 2. Materials and Experimental Detail

### 2.1. Materials

**Figure 1:**
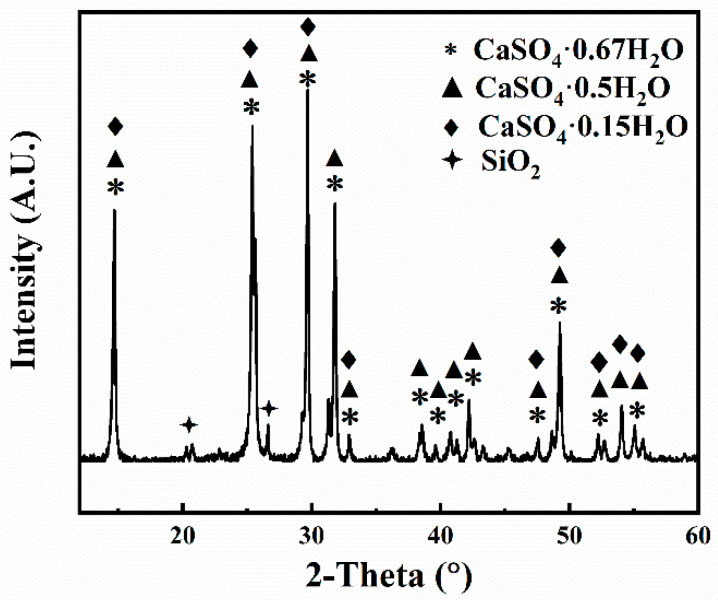
XRD spectrum of HPG.

**Figure 2:**
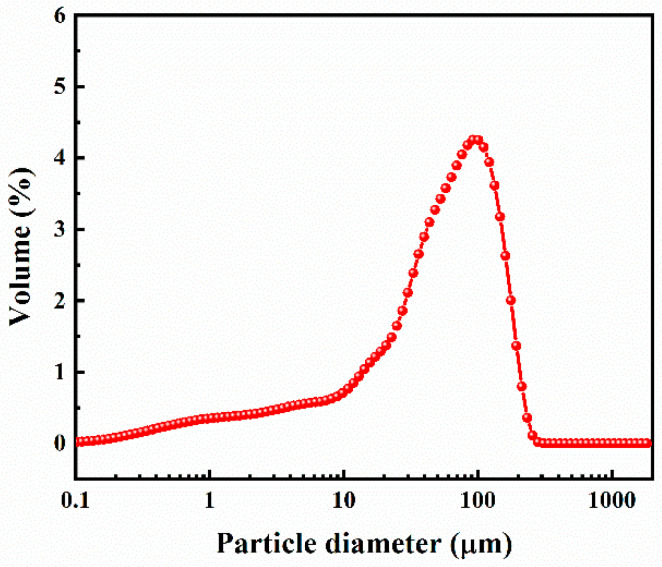
Particle size distributions of HPG.

HPG was obtained from Guizhou Wengfu Group Co., Ltd., Fuquan City, China. The X-ray diffraction (XRD) pattern of the HPG is displayed in Figure 1. The particle size distribution of the HPG is displayed in Figure 2. The particle size of the HPG ranged from 0.04 μm to 309.644 μm, and the mean particle size (d50) was 57.92 μm. The main crystal phases in HPG were CaSO_4_·0.5H_2_O and SiO_2_ (Figure 1). The chemical compositions of the PG are listed in Table 1, indicating that HPG contains 94.80 wt % CaSO_4_·0.5H_2_O (calculated from the contents of SO_3_ and CaO) and small amounts of SiO_2_, P_2_O_5_, and Al_2_O_3_. Radionuclides and soluble heavy metals in HPG can cause environmental problems, restricting its use in building materials. The heavy metal (Pb, Cd, Cr, and Hg) contents in the HPG were all less than the limit values of the Chinese standard (GB18582-2008) (Table 2).

Hydrophobic nanosilica (>99%, particle size: 15 nm, Brunauer–Emmett–Teller (BET): 300 ± 50 m^2^/g), n-hexane (97%), and octadecyltrichlorosilane (OTS) (95%) were purchased from Shanghai Macklin Biochemical Technology Co., Ltd. (Shanghai, China) Poly(methylhydrosiloxane) (viscosity: 15–40 mPa. s, 20 °C) was obtained from Jiuding Chemical (Shanghai, China) Technology Co., Ltd. H-PDMS (viscosity: 40 mpa. s) and dibutyl dilaurate (DBDL), which was used as the catalyst, were supplied by Shanghai Aladdin Biochemical Technology Co., Ltd (Shanghai, China).

### 2.2. Experimental Methods

#### 2.2.1. Preparation of PG Samples

PG samples (20 mm × 20 mm × 5 mm) with a water-to-gypsum ratio (w/c) of 0.6 were prepared in a mold. They were removed after the final set. Subsequently, the PG samples were dried at 50 °C for 24 h to avoid the influence of moisture on the treatment. At a room temperature of 25 °C, 5 g of NaOH and 0.3 g of Na_2_SO_4_ were added to 30 g of deionized water. After magnetic stirring for 10 min, the dried PG samples were immersed in an alkaline solution for 10 min. After drying at 55 °C for 24 h, hydroxylated PG (OH-PG) was obtained.

#### 2.2.2. Fabrication of Hydrophobic PG Sample

The hydrophobic coating agents were prepared and modified following Jadav’s report [29]. A mixture of H-PDMS and PMHS, acting as an in situ reaction polymer, and DBTDL as a catalyst was dissolved in 30 g of hexane solvent and stirred magnetically at 40 °C for 60 min to create a surface treatment agent called P-1. The mass of the H-PDMS was 10% of the mass of the hexane solvent; the mass ratio of the H-PDMS to PHMS was 10:1 and the amount of catalyst was 1% of the total polymer mass.

Similar to the method used above, the H-PDMS-PMHS solution was mixed with octadecyltrichlorosilane (OTS), constituting 5% of the solvent mass. This surface treatment agent was called P-2. Then, 0.6 g (2% solvent mass) of hydrophobic nanoparticles was added to the H-PDMS-PMHS-OTS solution, which was identified as P-3 and ultrasonically dispersed for 30 min.

**Figure 3:**
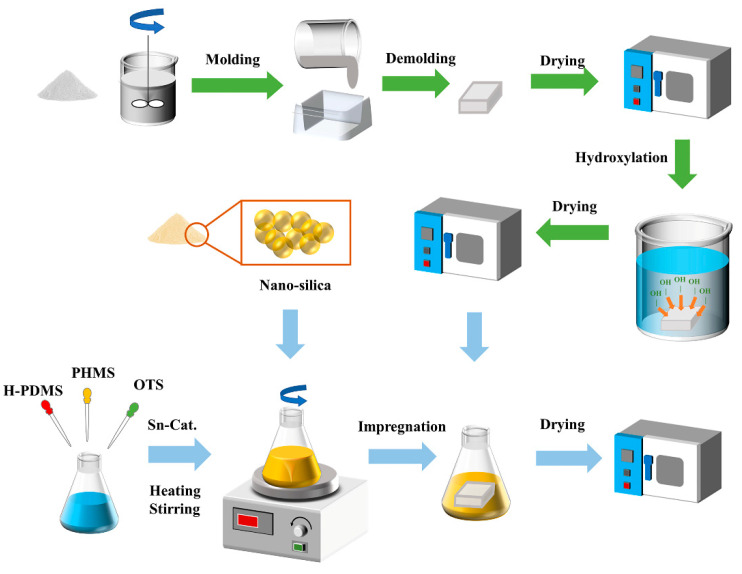
Experimental processing diagram.

The hydrophobic coating deposited on the PG surface was prepared using the impregnation method. The samples were then dried at 50 °C for 24 h to obtain PG with hydrophobic coatings. The hydrophobic PG prepared after 90 min in the surface treatment agents P-1, P-2, and P-3 was called PGH-1, PGH-1, and PGH-3, respectively. The entire experimental procedure is illustrated in Figure 3.

### 2.3. Sample Characterization

The phases, oxides, and size distribution of the HPG were analyzed using XRD (Ultma IV, Rigaku, Japan), an X-ray fluorescence spectrometer (ARL Perform’X, Thermo Scientific, USA), and a laser granularity instrument (Mastersizer 2000, Malvern Panalytical company, Nottingham, United Kingdom), respectively. A 3D optical profilometer (ContourGT-X3, Bruker, Saarbrücken, Germany) was used to analyze the surface roughness and morphology. Dry 20 mm × 20 mm × 5 mm original PG samples were polished with 240# sandpaper to remove impurities and construct a desired surface roughness for the hydrophobic treatment. The contact angle (10 μL deionized water) of the different sample surfaces was measured using an optical contact angle tester (JC2000D1, Shanghai Zhongchen digital technology Equipment Co., LTD, Shanghai, China). The surface elements of the different samples were examined using XPS (K-Alpha instrument, Thermo Scientific, Waltham, Massachusetts, MA, USA). All binding energies were revised using the C1 peaks of contaminated carbon at 284.8 eV. The micromorphologies of the samples were characterized using FTIR (Sigma 300, ZEISS, Oberkochen, Baden-Wurtberg, Germany). The structural information of the organic components was analyzed using attenuated total reflection FTIR (iS50, Nicolet, Madison, Wisconsin, USA). The sample powder was compressed with potassium bromide into tablets for the test. The scanning range of the FTIR was 400–4000 cm^−1^.

A TG 209 F3 Nevio Thermogravimetric Analyzer from NETZSCH Instruments was used to measure the mass of the sample as a function of temperature (or time) under programmed temperature control. The test gas atmosphere was nitrogen, the specific test temperature range was 30–650 °C, and the temperature increase rate was 10 °C/min. The surface grafting rate of the PG was calculated using the following equation:

Grafting rate = (*W*_A_ − *W*_B_)/*W*_C_, (1)

where *W*_A_ is the weight loss value of the PGH-1 when heated to 600 °C, *W*_B_ is the weight loss value of the PG or OH-PG when heated to 600 °C, and *W*_C_ is the residual weight of the PGH-1 when heated to 600 °C.

### 2.4. Stability and Durability Tests

An abrasion test was performed using SiC sandpaper (400# and weight of 100 g). HCl and NaOH were used to prepare solutions with different pH values at room temperature. The chemical stability of the superhydrophobic surfaces were tested by evaluating the CA values in solutions with different pH values (1–14). The adhesion test was performed using tape. The entire surface of the superhydrophobic coating was taped each time, and the tape was pulled up quickly. This process was repeated 100 times.

## 3. Results and Discussion

### 3.1. The Effect of Surface Hydroxylation on the Properties of PG

#### XRD and SEM Analysis

**Figure 4:**
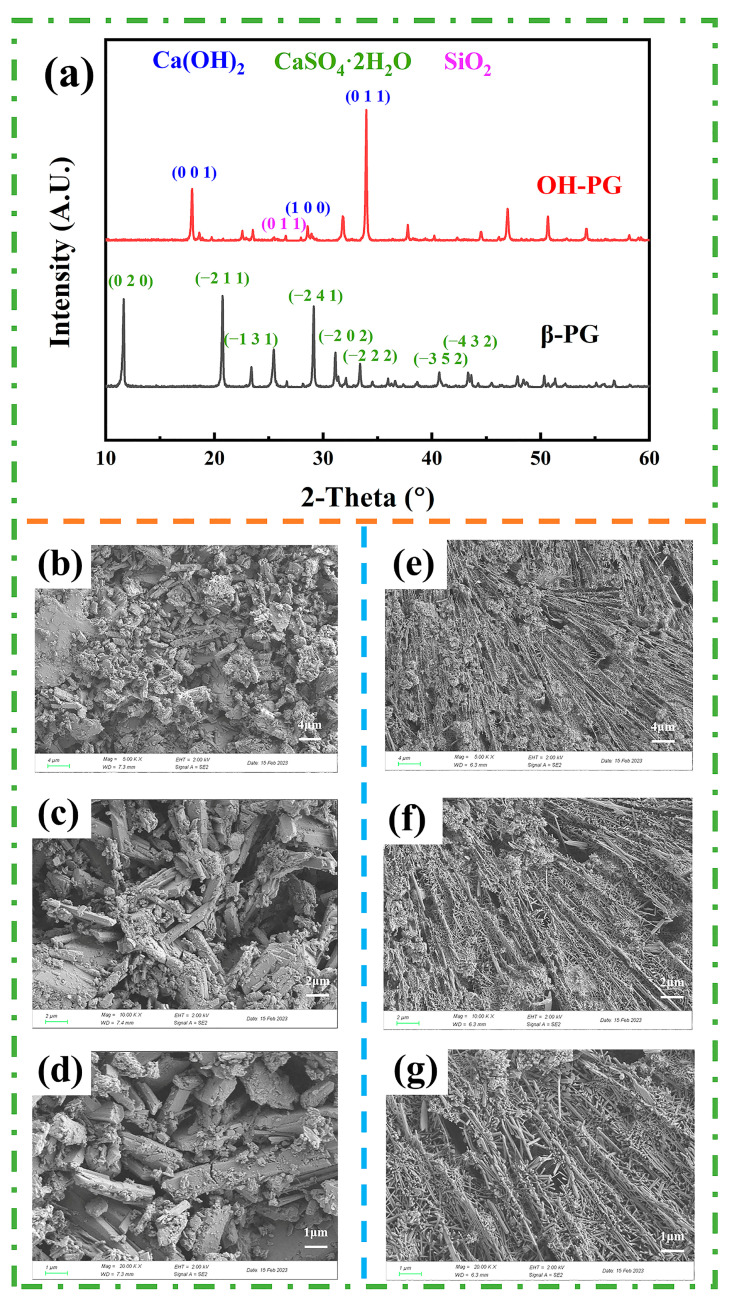
(**a**) XRD patterns of different phosphogypsum samples; (**b**–**d**) PG at different magnifications; (**e**–**g**) OH-PG at different magnifications; (**b**,**e**) 5000×; (**c**,**f**) 10,000×; (**d**,**g**) 20,000×.

A comparison of the XRD patterns before and after hydroxylation is shown in Figure 4a. The PG mainly consisted of CaSO4·2H_2_O (pdf# 72-0596, space group: C2/c) and SiO_2_ (pdf# 70-2536, space group: P3221). The 2θ values of 11.6°, 20.7°, 23.4°, 29.1°, 31.1°, 33.4°, 40.7°, and 43.4° observed in the XRD patterns of the PG correspond to CaSO_4_·2H_2_O (020), (-211), (-131), (-241), (-202), (-222), (-352), and (-432) respectively. The 2θ value of 26.8° is the main diffraction peak of the SiO_2_ (011) crystal plane.

Compared to the XRD pattern of the PG, the pattern of the OH-PG indicates new peaks at 2θ values of 18.3°, 28.9°, and 34.3°, which correspond to the diffraction peaks of the (001), (100), and (011) crystalline planes of Ca(OH)_2_, respectively. This was due to the reaction of NaOH with CaSO_4_, leading to the formation of Ca(OH)_2_ that was deposited on the PG surface. The Ca(OH)_2_ layer present on the PG surface also significantly increased the amount of surface hydroxylation. The PG surface hydroxylation reaction is as follows:

CaSO_4_ + 2NaOH ⇌ Na_2_SO_4_ + Ca(OH)_2_. (2)

As displayed in Figure 4b–d, the unhydroxylated PG surface indicates primarily long rod-like crystals of calcium sulfate dihydrate. After hydroxylation, the rod-like crystals on the PG surface changed to needle-like Ca(OH)_2_ after the reaction described in Equation (2) (Figure 4e–g). The increase in the aspect ratio of the OH-PG crystals, increase in the tiny pores between the crystals, and larger specific surface area provided more active reaction sites for the subsequent preparation of the polysiloxane hybrid nanoparticle coating on the PG-based material surface.

### 3.2. FTIR Analysis

**Figure 5:**
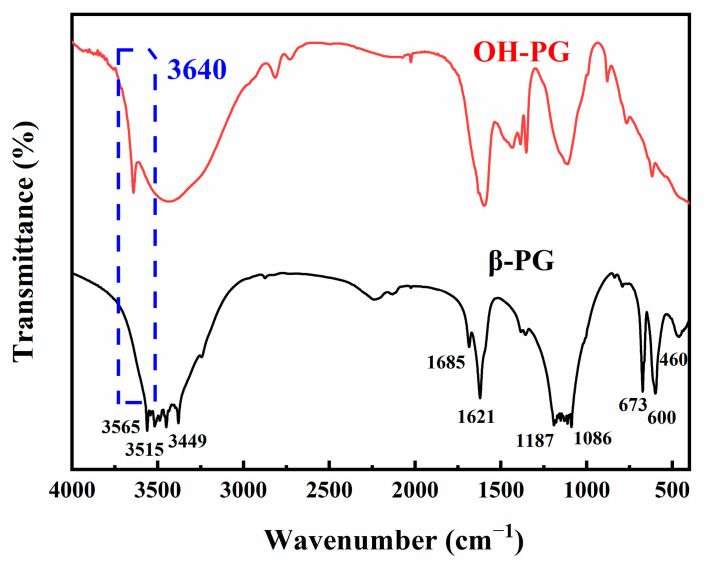
FTIR spectra of different phosphogypsum samples.

In the FTIR spectra displayed in Figure 5, the characteristic peaks of the hydration product calcium sulfate dihydrate appear in all the PG spectral lines. The adsorption peaks at 3415 and 3449 cm^−1^ are due to the symmetric stretching vibrations of the crystalline water in the CaSO_4_·2H_2_O. The peak at 3565 cm^−1^ can be attributed to the antisymmetric stretching vibration of the crystalline water. The peaks at 1686 and 1621 cm^−1^ are caused by the bending vibration of the crystalline water in the calcium sulfate dihydrate. The absorption peaks at 1187 and 1086 cm^−1^ can be attributed to the antisymmetric stretching vibration of the SO_4_^2−^. The peaks at 673 and 600 cm^−1^ are due to the antisymmetric bending vibrations of the SO_4_^2−^, and the peak near 460 cm^−1^ can be attributed to the symmetric bending vibrations of the SO_4_^2−^. However, the intensity of the stretching vibration peak at 3640 cm^−1^ is virtually absent, indicating that the number of hydroxyl groups on the PG surface was small.

The FTIR spectrum of the OH-PG indicates a strong hydroxyl stretching vibration peak at 3640 cm^−1^, indicating a significant increase in the number of hydroxyl groups on the OH-PG surface after hydroxylation. The formation of Ca(OH)_2_ on the surface of the OH-PG, deposited on the surface of the PG, led to a decrease in the intensity of the antisymmetric stretching vibration peaks of the SO_4_^2−^ at 1187 cm^−1^ and 1086 cm^−1^ and other characteristic peaks of the hydration product calcium sulfate dihydrate.

### 3.3. Thermogravimetric Analysis

**Figure 6:**
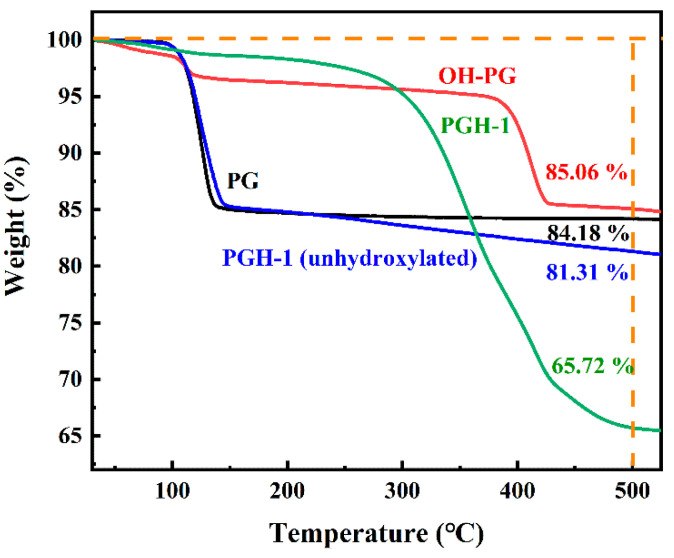
TG curves of different phosphogypsum samples.

The TG curves of the PG before and after hydroxylation are displayed in Figure 6. In the 0–100 °C range, all four PG samples indicate a small thermal weight loss due to the removal of the extrinsic water of the calcium sulfate dihydrate crystals. In the 100–130 °C range, the PG and PGH-1 (PG unhydroxylated and treated with H-PDMS/PMHS to prepare hydrophobic coatings) indicate significant thermal weight loss due to the removal of crystalline water. With a further increase in temperature, the onset of calcium sulfate decomposition temperature reached 1000–1200 °C [30]. Therefore, there was virtually no thermal weight loss in the 130–525 °C range for the PG samples. Conversely, PGH-1 indicated an additional 2.87% thermal decomposition compared to the PG sample, owing to the decomposition of the surface H-PDMS/PMHS siloxane chain segments.

At the end of the high-decomposition-rate phase at 350–500 °C, the weight loss of the OH-PG reached 14.94%, which can be attributed to the partial decomposition of Ca(OH)_2_ on the surface of the OH-PG. This further confirms the presence of a large number of hydroxyl groups on the surface after hydroxylation treatment. The weight loss of PGH-1 in the same temperature range was 34.28%, which was mainly due to the thermal weight loss of unreacted Ca(OH)_2_ and organic chain segments grafted on the OH-PG surface after the catalytic cross-linking of H-PDMS/PMHS. The grafting rate was calculated based on Equation (1). The grafting rate of the H-PDMS/PMHS on the PG surface after hydroxylation increased from 3.53% to 29.42%, indicating that the grafting rate of the hydrophobic siloxane chain segments on the PG surface significantly increased, likely owing to hydrogen bonding.

### 3.4. Surface Wettability Analysis

#### 3.4.1. Contact Angle and Adhesion Analysis

**Figure 7:**
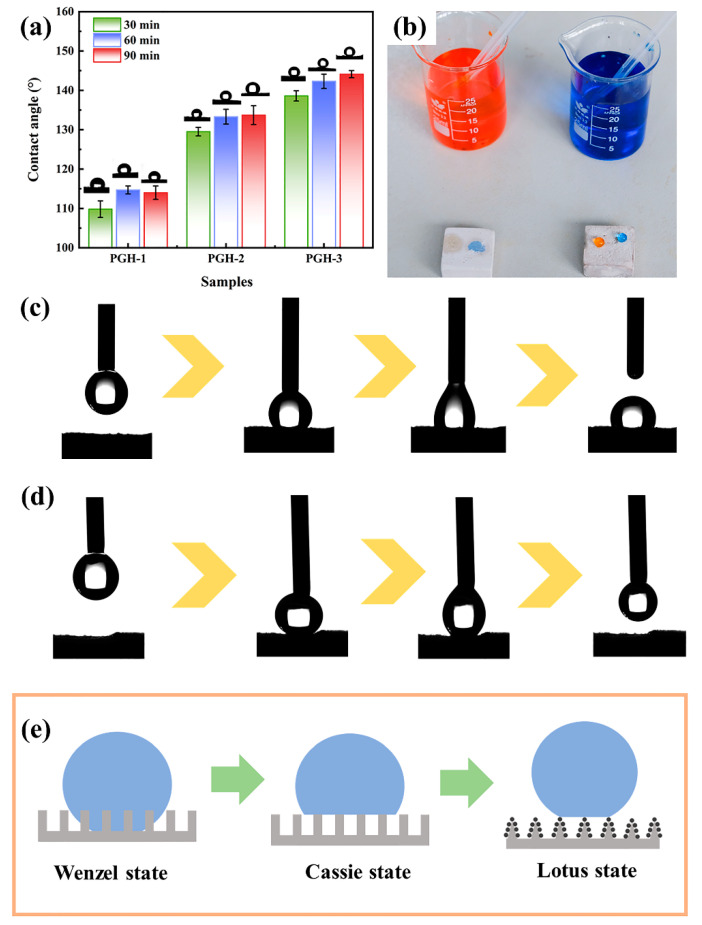
(**a**) Water contact angle of different phosphogypsum samples, (**b**) photographs of 0.1 mL aqueous solutions of methyl orange and methylene blue droplets on the PGH-3 hydrophobic surface, (**c**) the contact and departure process of the water droplet on PGH-2 surface, (**d**) the contact and departure process of the water droplet on PGH-3 surface, (**e**) surface wettability state analysis.

The effect of the impregnation time on the hydrophobicity of the different samples is displayed in Figure 7a. After 90 min of modification, the contact angles of the PGH-1 and PGH-2 were 114° and 133.7°, respectively, indicating that the cross-linking of OTS based on H-PDMS/PMHS further reduced the surface energy of the hydrophobic coating. The contact angle of PGH-3 (90 min impregnation) reached a maximum value of 144.1°, indicating that the hydrophobicity of the H-PDMS/PMHS/OTS coating under nano-SiO_2_ hybridization was further improved and approached a superhydrophobic state. The increase in hydrophobicity with impregnation time also contributed to the improvement in the hydrophobicity of the different samples. Photographs of the water droplets stained with methyl orange and methylene blue are displayed in Figure 7b. On untreated PG samples, water rapidly penetrated the PG hardened body and indicated superhydrophobicity, whereas on the surface of PGH-3 (90 min), the water droplets maintained their intact spherical shape.

Figure 7c,d compare the change in adhesion on the surface of the H-PDMS/PMHS/OTS coating with and without nano-SiO_2_ hybridization. The transition from high to low adhesion was achieved with the effect of nano-SiO_2_. This phenomenon can be explained by the schematic diagram in Figure 7e, where the low-surface-energy siloxane caused the surface to change from the Wenzel to the hydrophobic Cassie state, which eventually exhibits a low-surface-energy “lotus” effect due to the micro/nanostructure constructed by the hydrophobic nanoparticles.

The Young–Dupre equation was applied to quantify the adhesion work [31]:

*W*_LS_ = *ϒ*_L_ (1 + cos*θ*), (3)

where *W*_LS_ is the adhesion work performed to completely separate the water droplet from the hydrophobic surface, and L and S represent the liquid and solid phases, respectively. The surface tension of the water droplet *ϒ*_L_ is 72 mN·m^−1^ at room temperature of 25 °C, and *θ* represents the contact angle. The adhesion force of the superhydrophobic surface was calculated to be 0.013677 J·m^−2^ (CA = 144.1°), indicating very low adhesion on this hydrophobic surface.

#### 3.4.2. Self-Cleaning Properties

**Figure 8:**
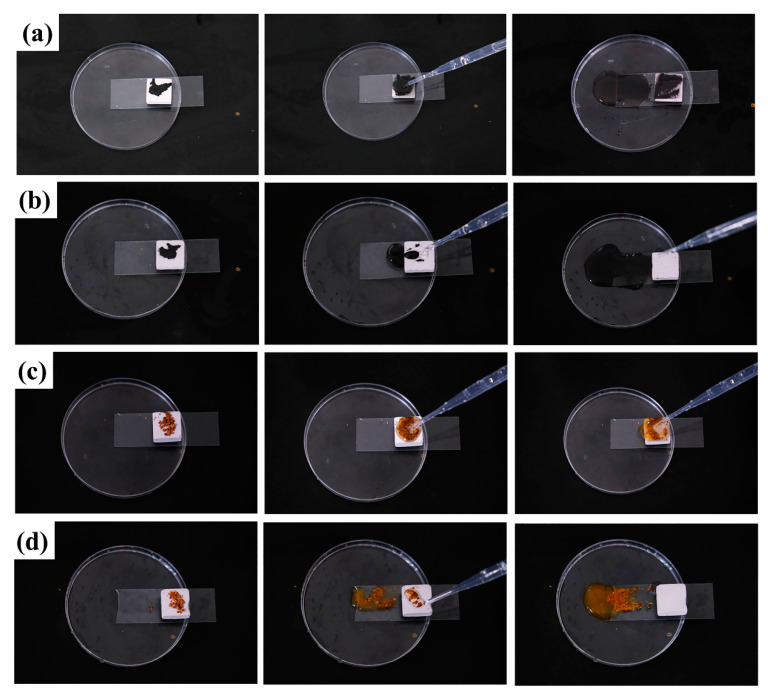
Self-cleaning performance test of the PG and PGH-3 (**a**,**b**): iron powder, (**c**,**d**): fine grains of sand stained with methyl orange.

As indicated in Figure 8, to simulate the antifouling and self-cleaning properties of the PG hydrophobic coating, iron powder and methyl-orange-stained fine grit were applied as stains on the sample surface. The self-cleaning properties of the hydrophobic surface were observed by dripping deionized water. As shown in Figure 8a,c, the untreated PG was placed tilted in a transparent plastic Petri dish and coated with iron powder and methyl-orange-stained fine grit, respectively. After dropping 1 mL of deionized water, the water droplets quickly wetted the surface and penetrated the interior of the pores due to the high water absorption and superhydrophobicity of PG. Iron powder and fine gravel were completely retained on the PG surface, with only a small amount of water flowing down from the inclined PG after the surface was completely saturated.

PG (PGH-3, 90 min) with a hydrophobic coating was used for comparison Figure 8b,d. After dripping deionized water, the water aggregated due to the high adhesion to iron powder and fine gravel. Moreover, the water droplets remained spherical under the action of the low-adhesion micro/nanostructured surface constructed by the low-surface-energy siloxane and hydrophobic nanosilica, and the stains on the PG material surface were quickly and completely removed by the “roll clean” effect of water droplets [32]. This demonstrates that PGH-3 had the same self-cleaning ability as lotus leaves found in nature.

### 3.5. Surface Microstructure Analysis

#### 3.5.1. Surface 3D Morphology and Roughness Analysis

**Figure 9:**
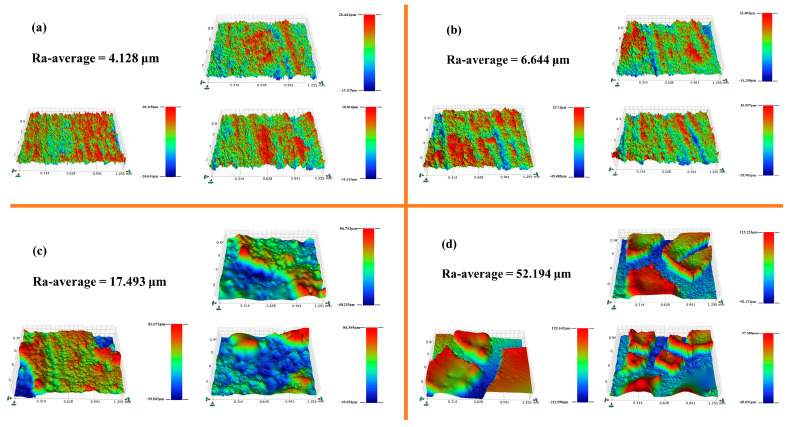
Three-dimensional morphology and roughness of the surface: (**a**) PG, (**b**) PGH-1, (**c**) PGH-2, (**d**) PGH-3.

Figure 9 and Table 3 present the 3D morphological images and roughness data for different PG surfaces. The average roughness of the untreated sample PG surface (Figure 9a) was 4.128 μm, and the substrate surface was relatively flat. The surface roughness of PGH-1, treated with H-PDM/PMHS, was marginally improved (Figure 9b). Conversely, the average roughness of PGH-2 significantly increased to 17.493 μm (Figure 9c), demonstrating irregularly interlaced bumps on the surface, indicating an undulating mound-like morphology. This suggests that the cluster-like protrusions formed by the long-chain alkyl groups were attached to the surface of the PG, enhancing its hydrophobicity. As seen in the 3D surface morphology image in Figure 9d, the surface coating exhibited a large dispersion with an average surface roughness reaching a maximum of 52.194 μm, which could be attributed to the loosening of the polymeric lattice structure of the hybridized nanosilica and the formation of particle agglomerates.

#### 3.5.2. Surface Morphology Analysis

**Figure 10:**
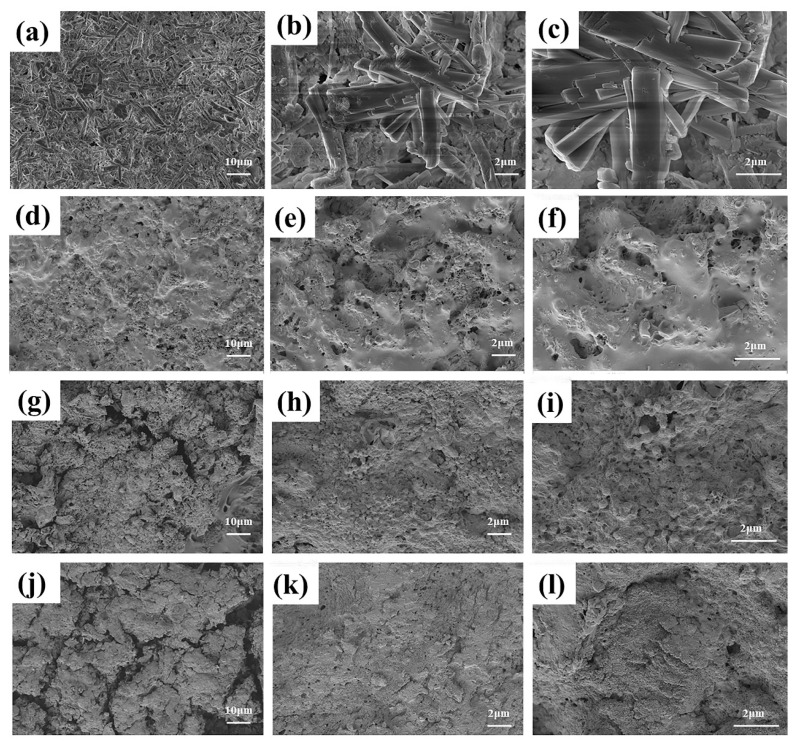
SEM images of specimen surfaces. (**a**–**c**) PG, (**d**–**f**) PGH-1, (**g**–**i**) PGH-2, and (**j**–**l**) PGH-3 at different magnifications. (**a**,**d**,**g**,**j**) 1000×, (**b**,**e**,**h**,**k**) 5000×, (**c**,**f**,**i**,**l**) 10,000×.

The microstructure of the surface of the HPG hydration products determined the macroscopic properties of the PG. In this section, the hydrophobic mechanisms of PGH-1, PGH-2, and PGH-3 are further explained from a microscopic perspective. This is achieved by analyzing the SEM images of the samples after 90 min of treatment by different methods. The control sample PG is displayed in Figure 10a–c. After the hydration of the HPG, calcium sulfate dihydrate was formed. Long rod-shaped crystals were lapped and interlaced with each other, and a large number of pores existed. Water could penetrate these pores to generate bidirectional pressure, which caused internal stress within the PG. Water could also dissolve the crystalline contact points of the PG-hardened body to make it recrystallize and reduce the strength [33,34]; hence, macroscopically, it indicates a reduced softening coefficient and a higher water absorption rate.

The microscopic morphology of the surface of the PGH-1 is displayed in Figure 10d–f. The surface of the PG crystals was completely covered by reticular material, indicating that the cross-linked H-PDMS/PMHS could form a hydrophobic coating on the surface of the PG through hydrogen bonding. As shown in Figure 10g–i, the addition of OTS further participated in the catalytic cross-linking between siloxanes, and the large number of clustered protrusions accompanied by the arrangement of a large number of hydrophobic -CH_3_ groups acted as a barrier to prevent water contact with the calcium sulfate dihydrate crystals. The hybridization of nanosilica further reduced the surface energy of the surface coating and filled the pores in the calcium sulfate dihydrate crystals and polymer network structure (Figure 10j–l).

### 3.6. Surface Chemical Composition Analysis

#### 3.6.1. XPS Analysis

**Figure 11:**
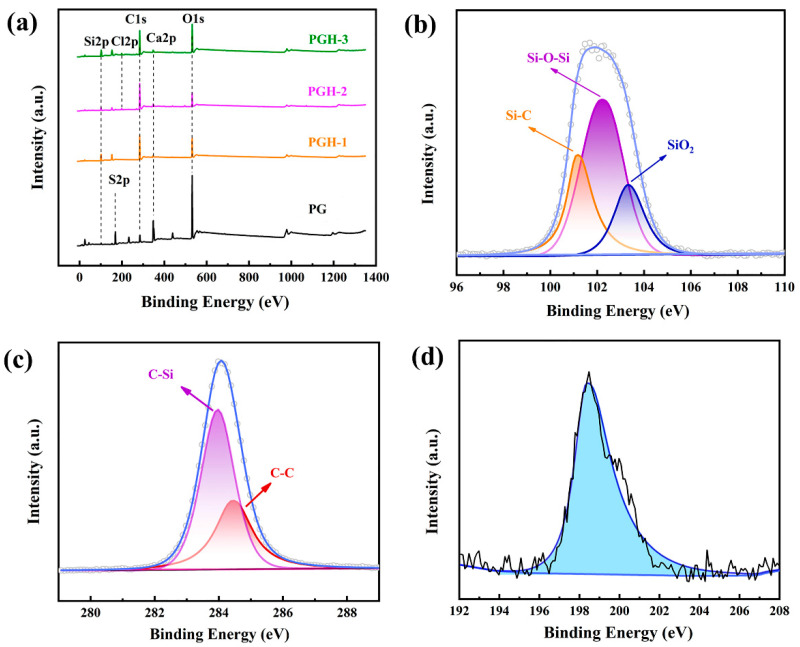
XPS spectrum of different samples. (**a**) Full spectrum, (**b**–**d**) Si 2p, C 1s, and Cl 2p of PGH-3 high-resolution spectrum.

The surface elements of the PG were qualitatively and quantitatively analyzed; the experimental results are displayed in Figure 11 and Table 4. The primary elements in the PG, PGH-1, PGH-2, and PGH-3 were O, C, S, Ca, Si, and Cl. Compared with that of PG (Si: 3.24%), the surface silicon content of the three hydrophobically treated PG species (PGH-1, PGH-2, and PGH-3) was significantly increased, reaching 18.42%, 9.19%, and 17.91%, respectively. The carbon content in the PG increased from 16.63% to 53.39%, 65.96%, and 48.82% in PGH-1, PGH-2, and PGH-3, respectively. The large increase in elemental Si and carbon content originated from the increase in the amounts of Si-O and -CH_3_ in the hydrophobic cross-linked network on the surface of the PG, and the -CH_3_ in the tail of the OTS molecule could also have increased the intensity of the C1s peak. The increase in elemental Cl content in the PGH-2 and PGH-3 originated from the partial Si-Cl of the incompletely reacted OTS.

From the high-resolution Si2p spectrum of PGH-3 displayed in Figure 11b, it can be observed that the absorption peaks of the Si-O-Si chain segment on the surface of the PG have an electron binding energy of 102.4 eV; the characteristic absorption peaks of SiO_2_ nanoparticles are at 103.98 eV and the characteristic absorption peaks of Si-C are at 101.2 eV [35,36,37]. The characteristic peak of PGH-3 at 284.6 eV binding energy in the C1s high-resolution spectrum of Figure 11c corresponds to the C-C bond in the main chain structure of the siloxane cross-linking network [38]; the characteristic peak at 283.60 eV corresponds to the Si-C [39]. The characteristic peak at 198.4 eV in the Cl2p spectrum of Figure 11d could correspond to the Si-Cl in the OTS [40].

#### 3.6.2. FTIR Analysis

**Figure 12:**
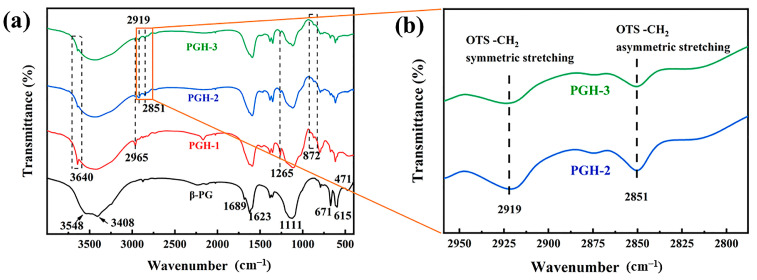
(**a**) FTIR spectra of different sample surfaces, (**b**) local enlarged image of FTIR spectra.

Comparisons of the FTIR spectra of the different PG samples are shown in Figure 12a. The characteristic peaks at 3548 and 3408 cm^−1^ for the blank control PG (CaSO_4_·2H_2_O) can be attributed to the symmetric stretching vibration of the crystalline water in the CaSO_4_·2H_2_O. The characteristic peaks near 1689 and 1623 cm^−1^ can be attributed to the bending vibration of the crystalline water in the calcium sulfate dihydrate. The characteristic peak at 1111 cm^−1^ can be ascribed to the antisymmetric stretching vibration of the SO_4_^2−^. The characteristic peaks at 671 and 615 cm^−1^ can be attributed to the antisymmetric bending vibration of the SO_4_^2−^. The characteristic peak near 471 cm^−1^ can be attributed to the symmetric bending vibration of SO_4_^2−^.

The above peaks also appeared in the hydrophobically treated PG samples, indicating that the coating did not change the basic structure of the PG. However, compared with the control curve of the PG, the other three IR spectra indicated new peaks due to the encapsulation of the hydrophobic coating; peaks at 2965, 1265, and 872 cm^−1^ are from the stretching vibration and symmetric deformation vibration of -CH_3_ in the siloxane and Si-O, respectively. Figure 12b displays the local FTIR spectra of the PGH-2 and PGH-3 in the range 2959–2788 cm^−1^. PGH-2 and PGH-3 have distinct vibrational peaks at 2851 cm^−1^ and 2919 cm^−1^, which correspond to the symmetric and asymmetric stretching vibrations of the OTS long-chain alkyl-CH_2_, respectively [41,42,43,44]. It is hypothesized that the -C_18_H_37_ long alkyl chains in the OTS successfully participated in the cross-linking reaction, further increasing the amount of hydrophobic -CH_3_ on the coating surface. FTIR spectra indicated the presence of closely arranged polymer hydrophobic networks on the PG surface, which reduced the surface energy of the PG and provided the necessary conditions for hydrophobic modification.

### 3.7. Stability and Durability Analysis

**Figure 13:**
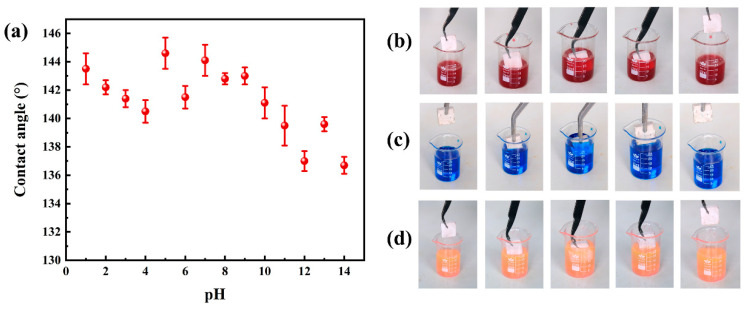
(**a**) Chemical stability of the PGH-3 surfaces, (**b**–**d**) photographs of the PGH-3 immersed in methyl-orange-dyed HCl solution (PH = 1), MB-dyed deionized water (PH = 7), and rhodamine 6G-dyed NaOH solution (PH = 14), respectively.

Chemical stability is a key factor for evaluating the hydrophobicity of PG surfaces. To demonstrate the ability of PGH-3 to maintain hydrophobicity under a range of acid- and alkali corrosion conditions, the contact angle of PGH-3 was studied in different pH (pH = 1–14) solutions, where the acidic solution stained with methyl orange changed from orange to red. As indicated in Figure 13a, the contact angle was greater than 140° in the 1–10 pH range, thus maintaining acceptable hydrophobic properties. At pH = 11–14, the contact angle decreased to below 140°; however, it remained greater than 136°, inferring that this change was caused by the partial dissolution of silica nanoparticles in the alkaline medium [45,46,47].

As indicated in Figure 13b–d, the surface of the PGH-3 remained dry after dipping it into staining solutions with different pH values and lifting it out with tweezers, which also proved the excellent chemical stability of PGH-3. After PGH-3 was immersed in the dyed solution, the “mirror” effect appeared due to the low surface energy of the surface coating. This effect was caused by the “air valley”, i.e., the air layer, between the H-PDMS/PMHS/OTS/SiO_2_ nanoparticles and air. It blocked further water contact with the PG surface [48]. According to the Cassie–Baxter theory [49], the air layer existing between a liquid and solid interface with low surface energy generates a reverse Laplace pressure, resulting in droplets suspended on the rough solid surface. The thicker the air layer between the solid and liquid interface, the easier it is to form a discrete solid–liquid–gas three-phase contact interface, which requires less energy for droplet roll-off and is more conducive to the construction of hydrophobic surfaces.

**Figure 14:**
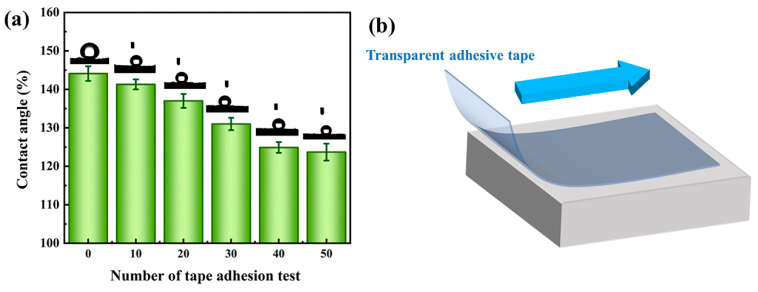
(**a**) Variation of contact angle with number of tape adhesion tests (**b**) schematic diagram of adhesive tape adhesion test.

Tape adhesion and peeling test is an effective method for testing the mechanical strength of hydrophobic coatings. Figure 14b displays a schematic of the experimental procedure. First, the tape was gently pressed to adhere to the PGH-3 surface. Subsequently, a weight was rolled over the part to be tested at a smooth and uniform speed. Finally, the contact angle was measured after the tape was peeled off and the surface was lightly blown using an ear wash ball. As indicated in Figure 14a, after 50 adhesion experiments, the PGH-3 surface maintained a contact angle of 123.7°.

**Figure 15:**
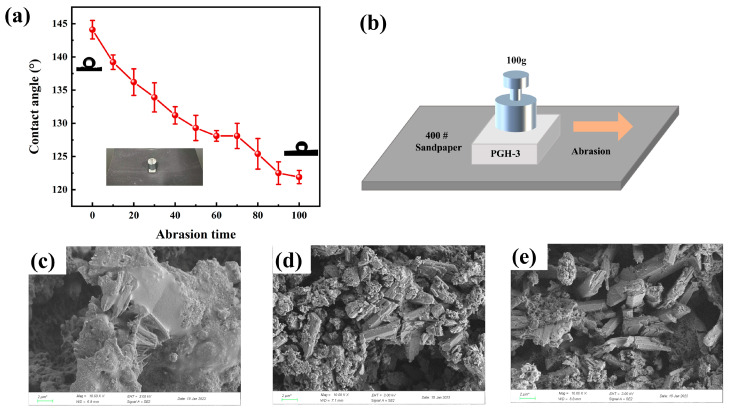
(**a**) CA of the PGH-3 with abrasion time, (**b**) schematic diagram of surface abrasion test, (**c**–**e**) SEM image of the 0-, 50-, and 100-times abrasion morphology of PGH-3, 10,000×.

The experimental wear process is shown in Figure 15b. To further investigate the wear resistance of the surface hydrophobic coating, a 100 g weight was placed on PGH-3 and moved at a speed of 5 cm/s on a 400# sandpaper surface; each 20 cm movement was recorded as one instance. Microscopic morphological changes in the wear process were observed and analyzed in combination with the change in the contact angle with the number of wear cycles (Figure 15c–e).

In the initial state without wear (Figure 15c), the PG surface was completely covered by the siloxane-hybridized nanosilica structure, and the long rod-like crystals of calcium sulfate dihydrate were completely encapsulated, resulting in a contact angle of 144.1°. After 50 wear cycles (Figure 3, Figure 4, Figure 5, Figure 6, Figure 7, Figure 8, Figure 9, Figure 10, Figure 11, Figure 12 and Figure 13d), the contact angle was further reduced to 129.3° due to the destruction of the hybridized aggregated state structure and partial exposure of the long rod-like crystals to air. After 100 wear cycles (Figure 15e), the micro-nano rough structure and hydrophobic cross-linked network on the surface of PGH-3 generated extremely high local pressure under mechanical loading, which caused the PG surface cover layer to gradually disappear and present staggered stacked gypsum crystals. Nevertheless, because of the distribution of hydrophobic silica particles in the microstructural pores, the surface of the gypsum crystals retained siloxane-hybridized nanosilica spines. Furthermore, the surface contact angle was maintained at 121.9° owing to the distribution of hydrophobic silica particles in the microstructure pores and the retention of spiny nanoprojections of siloxane-hybridized silica on the surfaces of the gypsum crystals.

### 3.8. Mechanism Analysis

**Figure 16:**
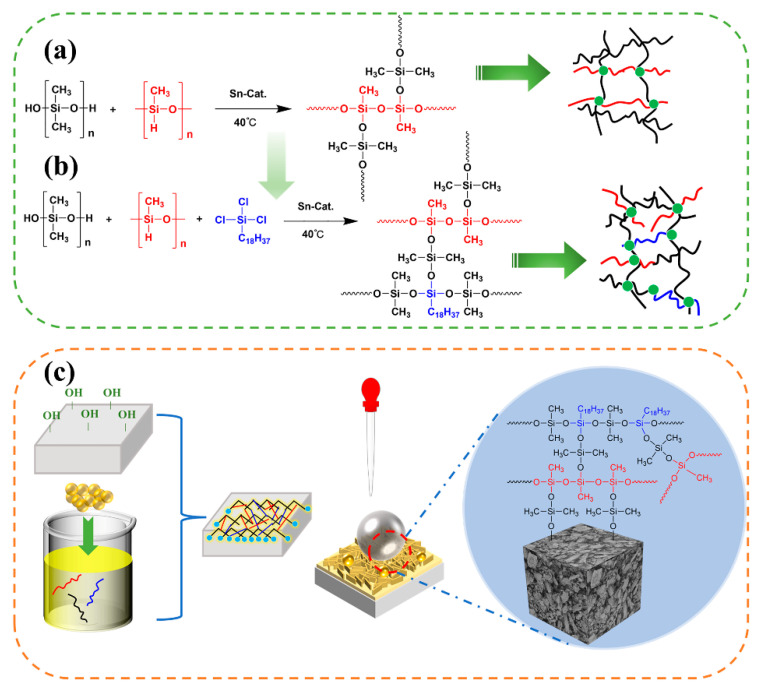
Schematic diagram of the chemical reaction. (**a**) Cross-linking reaction of PMHS and H-PDMS, (**b**) cross-linking reaction of PMHS, H-PDMS, and OTS, (**c**) hydrophobic mechanism of PGH-3 surface.

As shown in Figure 16a, the condensation reaction of the terminal hydroxyl group of H-PDMS with the Si-H of PMHS was catalyzed by organotin. A suitable amount of OTS is involved in the double cross-linking reaction between siloxanes, and the three Si-Cl bonds of OTS serve as reaction sites to react with the Si-OH group of H-PDMS to form Si-O-Si (Figure 16b). If OTS is not cross-linked with siloxane in the presence of a catalyst, it will hydrolyze in the environmental system and form aggregated silica nanoparticles; hence, OTS should not be used in excess [50]. The reaction equation for the hydrolysis of OTS to produce an isolated silanol structure is as follows:

C_18_H_37_SiCl_3_ + 3H_2_O→C_18_H_37_Si(OH)_3_ + 3HCl. (4)

During the above in situ reaction, organic/inorganic hybridization was performed by adding nanosilica, and hydroxylated PG was impregnated into this surface treatment agent. Through hydrogen bonding between the hydroxyl groups, the cross-linked polysiloxane hydrophobic network was attached to the PG surface, and the large amount of hydrophobic CH_3_ and rough surface constructed by the nanoparticles together caused the transformation of the originally completely hydrophilic PG into hydrophobic (Figure 16b). The PGH-3 surface used a micro/nano- rough structure and low surface energy to trap air and maintain the spherical shape of the droplets, while generating a low solid–liquid contact interface, thereby achieving a Cassie–Baxter state and ultimately presenting a contact angle of 140° or greater.

## 4. Conclusions

H-PDMS/PMHS/OTS hybrid nanosilica hydrophobic coatings were prepared on PG surfaces using the sol–gel and impregnation methods. The surface wettability, self-cleaning properties, microscopic morphology, surface roughness, surface chemical information, stability, and durability of the coatings on the PG samples after different hydrophobic treatments were investigated. The results of this study can be summarized as follows.

1. The maximum contact angle of the PGH-3 surface was 144.1°, with low adhesion to water droplets and acceptable self-cleaning performance. From the surface 3D morphology and SEM images, it was observed that the surface micro- and nano-hydrophobic networks with a certain roughness formed a hydrophobic coating on the PG.
2. XPS and FTIR tests demonstrated that the carbon and silicon elements on the PGH-3 surface increased to 48.82% and 17.91%, respectively, and the hydrophobic network of siloxane generated by the in situ reaction attached to the surface of the PG through hydrogen bonding such that the PG, which was originally completely hydrophilic, exhibited hydrophobicity.
3. The PGH-3 sample exhibited excellent chemical stability, and the contact angle could be maintained greater than 135° under strongly acidic or strongly alkaline conditions. After 50 tape-bonding tests, the contact angle remained at 123.7°. After 100 wear cycles, the contact angle remained at 121.9°.

Therefore, H-PDMS/PMHS/OTS hybrid nanosilica hydrophobic coatings prepared by the sol–gel method on the surfaces of PG-based materials have substantial potential for applications in the field of building materials. This method offers an effective means of utilizing gypsum-based solid waste with high added value and reduces environmental pollution problems caused by the phosphorus chemical industry.

## Figures and Tables

**Table 1:** Hemihydrate phosphogypsum composition.

Composition	Content/wt %	Composition	Content/wt %
SO_3_	58.620	TiO_2_	0.075
CaO	36.180	SrO	0.057
SiO_2_	2.340	BaO	0.046
P_2_O_5_	0.999	Y_2_O_3_	0.007
Al_2_O_3_	0.748	ZrO_2_	0.006
Fe_2_O_3_	0.532	Ar	0.004
MgO	0.157	Lu_2_O_3_	0.002
Na_2_O	0.130	CuO	0.002
K_2_O	0.096	NiO	0.001

**Table 2:** Soluble heavy metals in HPG.

	Demand of Standards	Test Results
Heavy metals	Pb ≤ 90 mg/Kg	Pb = 3.52 mg/Kg
	Cd ≤ 90 mg/Kg	Cd = 1.05 mg/Kg
	Cr ≤ 90 mg/Kg	Cr = 11.54 mg/Kg
	Hg ≤ 90 mg/Kg	Hg = 5.64 mg/Kg

**Table 3:** Surface roughness of different phosphogypsum samples.

Scheme 1	Ra-1/μm	Ra-2/μm	Ra-3/μm	Ra-Average/μm
PG	4.523	4.218	3.642	4.128
PGH-1	6.105	6.413	7.414	6.644
PGH-2	14.754	21.568	16.156	17.493
PGH-3	29.077	43.524	83.981	52.194

**Table 4:** Contents of each element in phosphogypsum samples.

Samples	C 1s/%	Ca 2p/%	O 1s/%	S 2p/%	Si 2p/%	Cl 2p/%
PG	16.63	11.79	54.28	13.34	3.24	0.72
PGH-1	53.39	1.07	25.05	1.7	18.42	0.37
PGH-2	65.96	2.2	18.84	1.72	9.19	2.09
PGH-3	48.82	1.61	28.41	1.54	17.91	1.71

## Data Availability

The data that were used are confidential.
